# Retraction: Superior efficacy of the antifungal agent ciclopirox olamine over gemcitabine in pancreatic cancer models

**DOI:** 10.18632/oncotarget.28601

**Published:** 2024-07-10

**Authors:** Chrysovalantou Mihailidou, Pavlos Papakotoulas, Athanasios G. Papavassiliou, Michalis V. Karamouzis

**Affiliations:** ^1^Molecular Oncology Unit, Department of Biological Chemistry, Medical School, National and Kapodistrian University of Athens, 11527 Athens, Greece; ^2^2nd Department of Medical Oncology, Theagenion Hospital, 54007 Thessaloniki, Greece; ^3^First Department of Internal Medicine, Laiko Hospital, Medical School, National and Kapodistrian University of Athens, 11527 Athens, Greece

**This article has been retracted:** Multiple instances of internal image duplication, as well as images that appear in earlier published papers, have been discovered in this article. Specifically, Figures 2B, 3, 4A, 4B, 5A, 6B, and 6C all have western blot bands image duplications. In addition, Figure 3 contains a western blot from Figure 7 of a previously published paper of the same group of authors [1]. Figure 5 also contains identical data from another previously published paper of the same group [2]. In light of these issues, the journal requested clarification regarding these images from the authors. The data and comments provided to Oncotarget did not resolve the concerns about the integrity and reliability of the reported data. Therefore, with the agreement of all authors except the first author, Chrysovalantou Mihailidou, the Oncotarget Editors retract this article.

Original article: Oncotarget. 2018; 9:10360–10374. 10360-10374. https://doi.org/10.18632/oncotarget.23164
